# How health risk factors affect inpatient costs among adults with stroke in China: the mediating role of length of stay

**DOI:** 10.1186/s12877-024-04656-4

**Published:** 2024-02-05

**Authors:** Xin Cao, Ruyu Li, Weiwei Tang, Wenjun Wang, Jingya Ji, Chengjie Yin, Luyao Niu, Yuexia Gao, Qiang Ma

**Affiliations:** 1 Department of Health Management, School of Public Health, Nantong University, Nantong, Jiangsu Province 226019 China; 2 Institute for Health Development, Nantong University, Nantong, Jiangsu Province 226019 China; 3 School of Health Policy and Management, Nanjing Medical University, Nanjing, Jiangsu Province 211166 China; 4 Affiliated Hospital, Nantong University, Nantong, Jiangsu Province 226019 China

**Keywords:** Stroke, Risk factors, Inpatient costs, Length of stay, China

## Abstract

**Background:**

As stroke has become the leading cause of death and disability in China, it has induced a heavy disease burden on society, families, and patients. Despite much attention within the literature, the effect of multiple risk factors on length of stay (LOS) and inpatient costs in China is still not fully understood.

**Aim:**

To analyse the association between the number of risk factors combined and inpatient costs among adults with stroke and explore the mediating effect of LOS on inpatient costs.

**Methods:**

A retrospective cross-sectional study was conducted among stroke patients in a tertiary hospital in Nantong City from January 2018 to December 2019. Lifestyle factors (smoking status, exercise), personal disease history (overweight, hypertension, dyslipidemia, diabetes mellitus, atrial fibrillation), family history of stroke, and demographic characteristics were interviewed by trained nurses. Inpatient costs and LOS were extracted from electronic medical records. Hierarchical multiple linear regression models and mediation analysis were used to examine the direct and indirect effects of the number of risk factors combined for stroke on inpatient costs.

**Results:**

A total of 620 individuals were included, comprising 391 ischaemic stroke patients and 229 haemorrhagic stroke patients, and the mean age was 63.2 years, with 60.32% being male. The overall mean cost for stroke inpatients was 30730.78 CNY ($ 4444.91), and the average length of stay (LOS) was 12.50 days. Mediation analysis indicated that the greater number of risk factors was not only directly related to higher inpatient costs (direct effect = 0.16, 95%CI:[0.11,0.22]), but also indirectly associated with inpatient cost through longer LOS (indirect effect = 0.08, 95% CI: [0.04,0.11]). Furthermore, patients with high risk of stroke had longer LOS than those in low-risk patients, which in turn led to heavier hospitalization expenses.

**Conclusions:**

Both the greater number of risk factors and high-risk rating among stroke patients increased the length of stay and inpatient costs. Preventing and controlling risk behaviors of stroke should be strengthened.

## Introduction

Globally, stroke is the second leading cause of death and disability, with over 13 million new cases annually, and the deaths and disabilities from stroke almost doubled during 1990–2016 worldwide [1]. In China, stroke has become a leading cause of death, long-term disability, and years of life loss [2, 3], and the disease burden has increased dramatically over the past 40 years [4, 5]. According to one study with 4,229,616 Chinese adults aged 40 years above from 31 provinces in mainland China conducted by the China Stroke Prevention Project Committee (CSPPC)(since 2011), the weighted prevalence of stroke increased from 2.28% in 2013 to 2.58% in 2019, with the average increase rate of 2.2% [4]. with approximately 2 million new cases annually, stroke may lead to a high and increasing disease burden, with the highest disability-adjusted life years lost of any disease in China [3].

With rapid population aging, urbanization, and lifestyle shifts, prevention and management of stroke in China remains an ongoing challenge [6]. The Global Burden of Diseases, Injuries, and Risk Factors Study (GBD) revealed that high systolic blood pressure, high body-mass index (BMI), high fasting plasma glucose (FPG), tobacco smoking, alcohol consumption, and low physical activity were the important and modifiable risk factors for stroke in 2019 worldwide [7], which contributing for large increase in the global burden of stroke Meanwhile, evidence in developed countries have shown that stroke is preventable and controllable [8, 9]. Currently, modifiable behaviors including physical activity have been proven to be crucial for the management of stroke [10].

To address the enormous challenge of stroke, the CSPPC launched the China Stroke High-risk Population Screening and Intervention Program (CSHPSIP) as a critical national project in 2011 [11]. In this screening project, eight high-risk factors for stroke were defined to detect those at risk among community-dwelling adults aged 40 years and above, including hypertension, diabetes, atrial fibrillation, dyslipidaemia, smoking, overweight or obesity, low physical activity, and family history of stroke. The CSHPSIP considers an individual with 3 or more risk factors to be at high risk of stroke. However, the relationship and underlying mechanism between the risk factors and inpatient costs of stroke have not been given attention in the last 10 years [11]. Most current studies have focused on the prevalence and disease burden of stroke and its attributing risk factors [12]. Therefore, this study aimed to explore the relationship between risk factors and stroke-related inpatient costs.

The economic burden of stroke patients is very heavy. In urban China, the weighted average annual direct medical cost per stroke was RMB 10,637 [95% CI = 10,435–10,840] (US$ 1682, [95% CI = 1650–1714]), with a full self-payment cost of RMB 3093 (US$ 489) of 29.04% of total stroke costs; moreover, the highest annual direct medical cost among haemorrhagic stroke patients was RMB 20,007 (US$ 3163), associated with the highest full self-payment percentage of 32.28% [13]. Previous studies have revealed that the length of stay (LOS) was an important factor affecting the inpatients costs in adults with stroke [14–18]. In China, a report conducted among ischaemic stroke inpatients from 121 hospitals in Beijing from March 1, 2012, to February 28, 2015, revealed that LOS, hospital level and pulmonary infection were key determinants of hospitalization expenses [19]; another retrospective study that collected records on stroke-related first hospitalizations in Shanghai from January 1, 2016, to December 31, 2019, showed that the main factors influencing hospitalization cost were LOS, hospital level, and whether surgery was performed [20]. Another report showed that factors affecting inpatient costs were age, day of the week of discharge, LOS, stroke subtype, other neurological disorders, renal failure, fluid and electrolyte disorders, and total number of comorbidities [21]. Moreover, LOS was an important common factor for hospital costs in different regions in China, and further studies should be encouraged to identify the appropriate and optimal LOS strategies for different diseases [22]. Medical insurance type is another noteworthy factor affecting healthcare expenditures. Total health costs among patients who had medical insurance were higher than those without any insurance [19].

In summary, few studies in China have explored the relationship between risk factors and inpatient costs, as well as the relationship between risk factors and LOS. The aim of our study was to evaluate (1) the relationship among the number of risk factors combined for stroke, LOS, and inpatient costs; (2) the potential mediating role of LOS in the association between stroke risk factors and inpatient costs; (3) the independent effect of the single risk factor for stroke and risk factors rating group on inpatient costs among adults with stroke.

## Methods

### Study design and participants

A retrospective and cross-sectional study was conducted among stroke patients in a tertiary hospital from January 2018 to December 2019. The hospital is the only designated hospital for the CSHPSIP in Nantong City, Jiangsu Province, PRC. The enrolment criteria of participants were: (1) discharged patients with ICD-10 codes I60-I69 and cerebrovascular disease as the main diagnosis; (2) normal communication skills, a high degree of cooperation, and strong compliance; (3) access baseline electronic medical record data; and (4) aged over 18 years. The exclusion criteria of participants were: (1) serious diseases or functional defects (e.g., mental illness, blindness, deafness, and dementia); and (2) emotional problems or traumatic experiences in the past 6 months (e.g., death of a relative, diagnosis of a terminal illness). Finally, 620 participants were included in this study.

This study followed the principles outlined in the Declaration of Helsinki and was approved by the institutional medical ethics committee of Nantong University, Jiangsu Province, PRC. Written informed consent was obtained from all patients, or an appropriate family member (in situations where the patient was disabled) to participate.

### Procedures

We assessed eight risk factors related to stroke, which were recommended and outlined by the China Stroke High-risk Population Screening and Intervention Program (CSHPSIP). First, physio­logical indicators including blood pressure, random blood glucose, electrocardiogram (ECG), total cholesterol (TC), triglycerides (TGs), high-density lipoprotein cholesterol (HDL-C), and low-density lipoprotein cholesterol (LDL-C) were measured. Second, questionnaires included sociodemographic characteristics (age, sex, body weight, height, marital status, social healthcare insurance status), lifestyle factors (history of smoking, physical inactivity), personal and family medical history (hypertension, dyslipidemia, diabetes mellitus, atrial fibrillation, transient ischemic attack [TIA], stroke) was face-to-face interviewed by trained nurses. These nurses involved in the survey were trained in the program and evaluated by theoretical and practical tests. Third, the hospitalization costs and length of stay of stroke patients were extracted from electronic medical records.

### Measures

#### Independent variables: risk factors, number of risk factors, and risk factor rating

In this study, we assessed stroke-related risk factors based on the guidelines of the CSHPSIP [11], including hypertension, atrial fibrillation or valvular heart disease, diabetes mellitus, smoking, dyslipidaemia, physical inactivity, obesity, and family history of stroke. Hypertension was defined as: (1) systolic blood pressure ≥ 140 mm Hg and/or diastolic blood pressure ≥ 90 mm Hg; (2) self-reported hypertension; (3) use of antihypertension medications. Atrial fibrillation was defined as a self-reported history of persistent atrial fibrillation or ECG results. Diabetes mellitus was defined as: (1) fasting plasma glucose ≥ 7.0 mmol/L; (2) self-reported diabetes mellitus; (3) use of oral hypoglycaemic agents or insulin injections. Dyslipidaemia was defined as (1) abnormal fasting plasma markers (triglycerides ≥ 2.26 mmol/L, total cholesterol ≥ 6.22 mmol/L, high-density lipoprotein cholesterol < 1.04 mmol/L, low-density lipoprotein cholesterol ≥ 4.14 mmol/L); (2) self-reported dyslipidaemia; (3) use of anti-dyslipidaemia medications. Smoking exposure was defined as continuous or cumulative smoking for more than six months in life and those who had quit smoking for more than six months at the time of the survey were excluded. Physical inactivity was defined as moderate and vigorous physical exercise performed for < 3 times per week and < 30 min each time. Moderate activities refer to activities that take moderate physical effort and make breathing somewhat harder than normal. Vigorous physical activities refer to activities that take hard physical effort and make breathing much harder than normal. Obesity was defined as body mass index (BMI) ≥ 28 kg/m^2^.

Furthermore, according to the Guideline of CSHPSIP, the individuals were divided into three categories as risk rating: high-risk, moderate-risk, and low-risk individuals for stroke.

High-risk individual: with three or more of the above-mentioned eight risk factors for stroke, or who had TIA or had a previous stroke.

Moderate-risk individual: with less than three of eight risk factors, but suffering from one of three chronic diseases, such as hypertension, atrial fibrillation, valvular heart disease, or diabetes mellitus.

Low-risk individual: with less than three risk factors and no chronic diseases such as hypertension, atrial fibrillation, valvular heart disease, or diabetes mellitus.

#### Dependent variable: inpatient costs

Inpatient costs were calculated by summing different kinds of hospitalization cost variables extracted from the subjects’ electronic medical records, including general basic therapy service fees, nursing fees, diagnosis fees, treatment fees, operation fees, medicine fees, materials fees, and other fees during hospitalization. The total stroke inpatient costs were natural log transformed before regression analysis because they are highly right-skewed.

#### Mediating variable: length of stay (LOS)

The LOS was collected from patients’ electronic medical records during their hospitalization for stroke.

#### Covariates

Covariates included age (< 50, 50–59, 60–69, 70–79, and ≥ 80 years), sex (male or female), marital status (single/unmarried or married), BMI (< 18.5, [18.5–24), [24–28), and ≥ 28 kg/m^2^) and medical insurance categories (Urban Employee Basic Medical Insurance (UEBMI), Urban Resident Basic Medical Insurance (URBMI), full self-payment, and other insurance).

### Statistical analysis

Stata software (v.15.0) and IBM SPSS Statistics (v.27.0) were used to analyze the data. First, the basic characteristics of participants were described using summary statistics, that is, means, standard deviations (continuous variables), frequency distributions, percentages (categorical variables), medians, and quartile ranges (variables that did not have a normal distribution). The differences in the variables were compared between the two stroke subtypes.

**Fig. 1:**
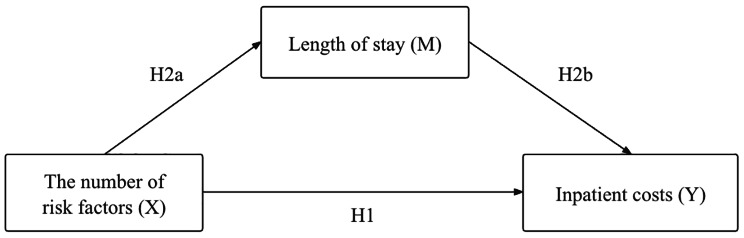
A hypothetical model of relationships among the number of risk factors, LOS and inpatient costs

Second, the association among the number of risk factors, LOS, and inpatient costs (see Fig. 1) was examined using mediation analysis based on principles from Baron and Kenny [23]. Hierarchical multiple linear regression models were used to examine the effects of the number of risk factors on inpatient costs and explore the mediating role of LOS in these associations. Model 1 examined the relationship between the number of risk factors (X) and inpatient costs (Y) (H1 in Fig. 1). Model 2 analyzed the relationship between the number of risk factors (X) and LOS (M) (H2a in Fig. 1), and Model 3 tested the direct effect of the number of risk factors (X) and LOS (M) on inpatient costs (Y) (H2b in Fig. 1). All the control variables were entered into these three models.

Third, Hayes (2015) SPSS macro program PROCESS was used to test the mediating role of LOS (M) in the relationship between inpatient costs (X) and the number of risk factors (Y). The mediation analysis was performed based on 5,000 bootstrapped samples, and bias-corrected 95% confidence intervals (CIs) were calculated. If the confidence interval values did not contain zero, the mediating effect was considered significant.

As additional analyses, we repeated the hierarchical multiple linear regression models to further explore the effect of different stroke risk rating groups on LOS and inpatient costs (Model 4, Model 5, and Model 6). Then, we repeated the hierarchical regression analysis to estimate the independent effect of the single risk factor on LOS and inpatient costs (Model 7, Model 8, and Model 9). All the models were adjusted for age, sex, BMI, marital status, medical insurance, and stroke subtype.

The results of hierarchical regression models were presented as coefficients and 95% confidence interval (CI). The *p* − value of < 0.05 was considered statistically significant.

## Results

### Participant characteristics

The demographic characteristics of the participants are summarized in Table 1. A total of 620 individuals were included, comprising 391 ischaemic stroke patients and 229 haemorrhagic stroke patients, and the mean age was 63.2 years, with 60.32% being male. The mean number of stroke risk factors was 2.38 (SD = 1.00). Additionally, according to the screening criteria of the high-risk population for stroke in the Chinese Stroke Screening and Prevention Project (CSSPP), 283 (45.65%) participants were identified at high risk of stroke, and 129 (20.81%) participants were in the low-risk group. The overall unadjusted mean inpatient costs were 30730.78 CNY ($ 4444.91), and the average length of stay (LOS) was 12.50 days (SD = 5.32). Comparisons between the two groups of stroke subtypes are shown in Table 1.

**Table 1:** The basic characteristics of participants

Variables	Ischaemic stroke n1 = 391	Haemorrhagic stroke n2 = 229	Total *n* = 620	x^2^ / t/Z
	n (%)	n (%)		
**Sex**				1.36
Male	229(58.57)	145(63.32)	374(60.32)	
Female	162(41.43)	84(36.68)	246(39.68)	
**Age** (years)	65.70 ± 12.98	59.00 ± 14.22	63.22 ± 13.83	5.979^***^
< 50	42(10.74)	52(22.71)	94(15.16)	33.86^***^
50–59	73(18.67)	67(29.26)	14(22.58)	
60–69	111(28.39)	51(22.27)	16(26.13)	
70–79	112(28.64)	42(18.34)	15(24.84)	
≥ 80	53(13.55)	17(7.42)	70(11.29)	
**Marital Status**				1.15
Single/unmarried	2(0.51)	3(1.31)	5(0.81)	
Married	389(99.49)	226(98.69)	615(99.19)	
**BMI** (kg/m^2^)	24.7 ± 3.1	24.6 ± 3.2	24.7 ± 3.1	1.2
< 18.5	8(2.05)	5(2.18)	13(2.10)	
18.5–23.9	148(37.85)	96(41.92)	24(39.35)	
24-27.9	185(47.31)	103(44.98)	28(46.45)	
≥ 28	50(12.79)	25(10.92)	75(12.10)	
**Medical insurance**				16.95^***^
UEBMI	127(32.48)	69(30.13)	196(31.61)	
URBMI	210(53.71)	99(43.23)	309(49.84)	
Full self-payment	46(11.76)	55(24.02)	101(16.29)	
Other	8(2.05)	6(2.62)	14(2.26)	
**Hypertension**				0.00
No	111(28.39)	65(28.38)	17(28.39)	
Yes	280(71.61)	164(71.62)	44(71.61)	
**Diabetes mellitus**				5.94^*^
No	274(70.08)	181(79.04)	45(73.39)	
Yes	117(29.92)	117(20.96)	16(26.61)	
**Atrial fibrillation**				27.27^***^
No	321(82.10)	221(96.51)	54(87.42)	
Yes	70(17.90)	8(3.49)	78(12.58)	
**Smoking**				0.64
No	182(46.55)	99(43.23)	28(45.32)	
Yes	209(53.45)	130(56.77)	33(54.68)	
**Dyslipidaemia**				1.20
No	323(82.61)	199(86.90)	52(84.19)	
Yes	68(17.39)	30(13.10)	98(15.81)	
**Physical inactivity**				7.14^**^
No	221(56.52)	104(45.41)	32(52.42)	
Yes	170(43.48)	125(54.59)	29(47.58)	
**Obesity**				1.66
No	338(86.45)	206(89.96)	54(87.74)	
Yes	53(13.55)	23(10.04)	76(12.26)	
**Family history of stroke**				0.50
No	385(98.47)	227(99.13)	61(98.71)	
Yes	6(1.53)	2(0.87)	8(1.29)	
**Inpatient costs** (RMB) (M(QR))	27551.64(21141.83)	38302.31(48341.60)	30730.78(27729.89)	-5.53^***^
**Log (Inpatient costs)** (Mean ± SD)	10.29 ± 0.69	10.63 ± 0.86	10.41 ± 0.77	-5.02^***^
**LOS** (days) (Mean ± SD)	11.74 ± 4.62	13.79 ± 6.13	12.50 ± 5.32	-4.40^***^
**Number of risk factors for stroke** (Mean ± SD)	2.44 ± 1.02	2.30 ± 10.7	2.38 ± 1.00	1.71^*^
**Stroke risk factor rating**				2.54
Low	75(19.18)	54(23.58)	129(20.81)	
Moderate	129(32.99)	79(34.50)	208(33.55)	
High	187(47.83)	96(41.92)	283(45.65)	

Note:

(1) *p < 0.05, **p < 0.01, ***p < 0.001

(2) Abbreviation: BMI: body mass index; UEBMI: Urban Employee Basic Medical Insurance; URBMI: Ubran and Rural Residents Basic Medical Insurance; LOS: Length of stay

### The number of risk factors and inpatient costs among adults with stroke

**Fig. 2:**
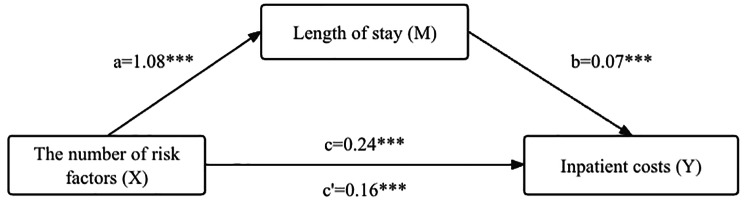
Direct effect and indirect effect of the number of risk factors for stroke on inpatient costs via length of stay. Unstandardized coefficients are shown in their path arrows

Hierarchical regression analysis was used to evaluate the relationship among the number of risk factors combined for stroke, LOS, and inpatient costs (see Table 2). Model 1 (in Table 2) showed that the number of risk factors was significantly and positively associated with inpatient costs (*β* = 0.24, *P* < 0.001, path c in Fig. 2) after adjusting for covariates, which explained 5.28% of the variance in inpatient costs, and Hypothesis 1 was supported. LOS was significantly predicted by the number of risk factors in Model 2 (*β* = 0.08, *P* < 0.001), which met the first requirement (path a in Fig. 2). When the independent variable, mediator, and control variables were entered into the equation model (Model 3 in Table 2), LOS was significantly and positively associated with inpatient costs (*β* = 0.07, *P* < 0.001), which met the second requirement (path b in Fig. 2). According to Baron and Kenny’s (1986) method, both criteria of the mediation model were met, indicating that LOS may act as a potential mediator, and Hypothesis 2 was supported. In addition, from Model 1 to Model 3, the regression coefficient for inpatient costs concerning the number of risk factors for stroke (from *β* = 0.24 to *β* = 0.16, *P* < 0.001) was reduced and was still significant (path c’ in Fig. 2), indicating that LOS partially mediated the relationship between the number of risk factors and inpatient costs.

**Table 2:** Hierarchical regression among number of risk factors for stroke, LOS, and inpatient costs

variables	Log (Inpatient costs)	Length of stay (LOS)	Log (Inpatient costs)
	Model 1	Model 2	Model 3
	Coefficient (95% CI)	Coefficient (95% CI)	Coefficient (95% CI)
**Independent variable**			
**Number of risk factors**	0.24(0.18,0.30)^***^	1.06(0.62,1.49)^***^	0.16(0.11,0.22)^***^
**Mediator**			
**LOS**			0.07(0.06,0.08)^***^
**Covariates**			
**Stroke subtypes** (ref: Ischaemic stroke)			
Haemorrhagic stroke	0.37(0.24,0.49)^***^	2.07(1.21,2.94)^***^	0.22(0.11,0.33)^***^
**Sex** (ref: Male)			
Female	0.23(0.10,0.35)^**^	-0.32(-1.22,0.58)	0.25(0.14,0.36)^***^
**Age** (ref: <50) (years)			
50–59	0.06(-0.13,0.26)	0.31(-1.06,1.69)	0.04(-0.13,0.21)
60–69	0.05(-0.15,0.24)	-0.83(-2.20,0.55)	010(-0.07,0.27)
70–79	-0.02(-0.22,0.18)	-0.35(-1.76,1.06)	0.01(-0.17,0.18)
≥ 80	-0.12(-0.36,0.12)	0.70(-0.98,2.37)	-0.17(-0.38,0.03)
**Marital Status** (ref: Single/unmarried)			
Married	-0.54(-1.20,0.12)	-5.60(-10.23, -0.97)^*^	-0.14(-0.72,0.43)
**BMI** (ref: <18.5)(kg/m2)			
18.5–23.9	0.20(-0.21,0.61)	1.87(-1.01,4.74)	0.07(-0.28,0.42)
24-27.9	0.09(-0.32,0.50)	1.25(-1.63,4.14)	0.00(-0.35,0.36)
≥ 28	-0.04(0.48,0.39)	1.67(-1.41,4.75)	-0.16(-0.54,0.22)
**Medical insurance** (ref: UEBMI)			
URBMI	0.15(0.02,0.28)^*^	0.50(-0.45,1.44)	0.11(-0.00,0.23)
Full self-payment	0.04(-0.14,0.22)	0.27(-0.99,1.52)	0.02(-0.13,0.18)
Other	0.03(-0.37,0.43)	0.30(-2.49,3.09)	0.01(-0.33,0.35)
**Adj R-squared**	12.74%	9.98%	34.29%

Note:

(1) *p < 0.05, **p < 0.01, ***p < 0.001

(2) The inpatient costs of stroke patients were naturally log-transformed in regression analysis

(3) All coefficients are unstandardized

(4) Abbreviation: BMI: body mass index; UEBMI, Urban Employee Basic Medical Insurance; URBMI, Urban Resident Basic Medical Insurance; LOS, length of stay

### Test for the mediating role of LOS

Table 3 reveals the results of the direct and indirect effects of the number of risk factors on inpatient costs through LOS in the mediation analysis by the bootstrap procedure. Figure 2 shows the regression coefficients for each pathway in the mediation model. First, the greater number of risk factors had a significant direct impact on increased inpatient costs (*β* = 0.16, Bootstrap 95%CI: [0.11,0.22]). Second, LOS mediated the effect of the number of risk factors on inpatient costs (*β* = 0.08, Bootstrap 95%CI: [0.04,0.11]), the mediating effect accounted for 33.33% of the total effect. These results highlighted that the greater number of risk factors among adults with stroke may incur longer LOS and more treatment, which may lead to higher total costs among inpatients.

**Table 3:** Mediaton effect of the number of risk factors for stroke on inpatient costs via LOS

Mediation path	Coefficient
	(Boot 95%CI)
Total effect (c)	0.24(0.18,0.30)
Direct effect (c’) Number of risk factors → Inpatient costs	0.16(0.11,0.22)
Indirect effect (a*b) Number of risk factors → LOS → Inpatient costs	0.08(0.04,0.11)
Adj-R^2^ = 0.33	

Note:

(1) 95% Boot CI, Bootstrap confidence interval with lower and upper limits

(2) Bold coefficients indicate significant associations with CIs that do not cross 0 (*P* value < 0.05)

(3) All the models were adjusted for age, sex, BMI, marital status, medical insurance, and stroke subtype

### Additional analysis

#### Stroke risk factor groups and inpatient costs

Table 4 reports the regression analysis of the effect of the risk factor rating group of stroke on LOS and inpatient costs. Model 5 showed that after controlling for covariates, compared with those in the low-risk group the total hospitalization cost among individuals in high-risk groups increased by 36% (β = 0.36, *P* < 0.01; Model 4 in Table 4), and had a 2.25-day (*β* = 2.25, *P* < 0.001; Model 5 in Table 4) increase in LOS Furthermore, when the risk factors rating variable and covariates entered in the model 6 in Table 4, LOS was significant and positive correlation with inpatient costs (*β* = 0.07, *P* < 0.001), indicating that high-risk factors in stroke patients had direct effect and indirect effect on total inpatient costs through LOS. In contrast, compared with low-risk patients, moderate-risk patients had a decrease of total inpatient costs and the absolute coefficient for inpatient costs was significant and elevated from Model 4 to Model 6 (from β=-0.20, *P* < 0.001 to β=-0.21, *P* < 0.01 in Table 4), but LOS was significantly and positively associated with inpatient costs, suggesting that moderate-risk group in stroke patients may have the masking effect on total inpatient costs, the underlying mechanism in these associations should be studied further.

**Table 4:** Hierarchical regression among stroke risk factor group, LOS, and inpatient costs

variables	Log (Inpatient costs)	Length of stay (LOS)	Log (Inpatient costs)
	Model 4	Model 5	Model 6
	Coefficient (95% CI)	Coefficient (95% CI)	Coefficient (95% CI)
**Stroke risk factor group** (ref: Low)			
Moderate	-0.20(-0.36, -0.03)^*^	0.13(-1.04,1.29)	-0.21(-0.35, -0.06)^**^
High	0.36(0.21,0.52)^***^	2.25(1.12,3.38)^***^	0.20(0.07,0.35)^**^
Mediator			
**LOS**			0.07(0.06,0.08)^***^
**Adj R-squared**	14.50%	8.20%	35.58%

Note:

(1) *p < 0.05, **p < 0.01, ***p < 0.001

(2) All coefficients are unstandardized

(3) Abbreviation: LOS, length of stay

(4) All the models were adjusted for age, sex, BMI, marital status, medical insurance, and stroke subtype

#### Single stroke risk factors and inpatient costs

We evaluated the contribution of each risk factor to LOS and inpatient costs after controlling for basic demographic characteristics and stroke subtype variables (see Table 5). The findings demonstrated that lack of physical exercise was significantly related to 1.47-day increase in LOS (Model 8 in Table 5) and a 27% increase in inpatient costs (Model 9 in Table 5), after adjusting for covariates. Smoking, obesity, comorbid diabetes mellitus, and atrial fibrillation among adults with stroke had only significantly contributed to increased inpatient costs, but were not related to LOS. However, hypertension only had the direct effect on LOS but not on inpatient costs Neither LOS nor inpatient cost was related to the family history of stroke.

**Table 5:** Hierarchical regression among single stroke risk factors, LOS, and inpatient costs

Variables	Log (Inpatient costs)	Length of stay (LOS)	Log (Inpatient costs)
	Model 7	Model 8	Model 9
	Coefficient (95% CI)	Coefficient (95% CI)	Coefficient (95% CI)
**Hypertension** (ref: no)			
yes	0.03(-0.11,0.16)	1.19(0.24,2.15)^*^	-0.05(-0.17,0.63)
**Diabetes mellitus** (ref: no)			
yes	0.26(0.13,0.40)^***^	0.81(-0.16,1.79)	0.19(0.07,0.31)^**^
**Atrial fibrillation** (ref: no)			
yes	0.22(0.04,0.41)^*^	1.14(-0.20,2.47)	0.11(-0.05,0.27)
**Smoking** (ref: no)			
Yes	0.30(0.11,0.49)^**^	0.072(-1.29,1.43)	0.29(0.13,0.45)^***^
**Dyslipidaemia** (ref: no)			
Yes	0.09(-0.08, 0.26)	-0.29(-1.53,0.93)	0.10(-0.05,0.24)
**Physical inactivity** (ref: no)			
Yes	0.36(0.23,0.48)^***^	1.47(0.57,2.37)***	0.27(0.16,0.38)^***^
**Obesity** (ref: no)			
yes	1.00(0.45,1.54)^***^	1.00(-2.94,4.95)	0.87(0.40,1.34)^***^
**Family history of stroke**(ref: no)			
yes	0.37(-1.14,0.88)	1.78(-1.94,5.49)	0.23(-0.22,0.67)
Mediator			
**LOS**			0.07(0.06,0.08)^***^
**Adj R-squared**	15.66%	4.98%	36.83%

Note:

(1) All coefficients are unstandardized

(2) *p < 0.05, **p < 0.01, ***p < 0.001

(3) Abbreviation: LOS, length of stay

(4) All the models were adjusted for age, sex, BMI, marital status, medical insurance, and stroke subtype

## Discussion

To our knowledge, this is the first retrospective study to explore the effect of the number of risk factors combined for stroke assessed in the guideline of CSHPSIP on inpatient costs and examine the mediating role of length of stay (LOS) between the number of risk factors combined for stroke and inpatient costs. Based on the conceptual model, this study had several main findings. First, we found that adults with stroke in China have a heavy inpatient burden. A greater number of risk factors among stroke patients was related to longer LOS and higher inpatient costs. Second, this study indicated that the LOS mediated the association between number of risk factors and inpatient costs. Finally, stroke risk factors rating group and physical inactivity had significantly direct effect and mediating effect on inpatient costs through LOS. These findings may enable to provide empirical evidence for reducing the disease burden of stroke patients.

### Comparison with other studies

The main contribution of this study is that the mediating role of LOS in the number of risk factors combined for stroke and inpatient costs have been verified in both hierarchical regression models and mediation analyses, which provides evidence for preventing and controlling risk factors for stroke so as to reduce stroke-related economic disease burden. Our finding indicated that as the number of risk factors including chronic diseases and overweight, elevated, both LOS and inpatient costs increased. Diabetes, hypertension, hyperlipidaemia, and atrial fibrillation were risk factors for stroke [24, 25]. The combination of these risk factors aggravated the admission condition of the patients, leading to an increase in LOS, more treatment, and more medicine utilization, which caused higher inpatient costs [26]. Diabetes, hyperlipidemia, and hypertension were common and poorly controlled in China, which contributed to an increased risk of stroke and a heavier disease burden. Moreover, our study further found that patients with more than three risk factors or a history of stroke had a longer length of stay and higher inpatient costs than those in the low-risk group.

LOS was the main factor affecting inpatient costs for stroke. The longer the LOS was, the higher the inpatient costs, which is consistent with current research [27]. LOS not only reflects the severity and recovery time of stroke patients but also embodies the comprehensive level of hospital and medical staff [28]. An inappropriate length of stay, on the one hand, would directly lead to bed costs, drug costs, nursing costs, and a series of basic cost increases; on the other hand, it would also affect the timeliness and convenience of treatment for other patients, reduce the working efficiency of the hospital and reduce social benefits. Therefore, reducing the number of risk factors combined for stroke could decrease the patients’ LOS, which in turn descend inpatient costs.

### Modifiable factors and inpatient costs

Additional analysis verified once again that comorbid with diabetes mellitus, atrial fibrillation, being overweight, and current smoking, lack of physical exercise had a remarkably impact on higher inpatient costs. Comorbid with hypertension and lack of physical exercise also significantly increased LOS. Poor lifestyles such as lack of physical exercise, smoking, and obesity are the leading risk factors and modifiable behaviors, which could cause the higher burden of cardiovascular diseases. A review reported that lifestyle factors including smoking, alcohol consumption, physical inactivity, a high-salt diet, and a high-fat diet played important roles in the occurrence and development of stroke [29]. A two-year randomized controlled intervention trial found that physical activity was associated with a reduced risk of stroke [30]. Thus, more effective public education and greater responsibilities of individuals should be strengthened to improve control of stroke risk factors.

Uncontrolled hypertension is the main risk factor for cerebral infarction and cerebral haemorrhage, management of hypertension is the essential public strategy for reducing the risk of incidence and mortality of stroke [31]. Although improvement in patient awareness and the treatment of hypertension and diabetes in China, the proportion of individuals whose hypertension and diabetes were controlled was less than 50%, which was lower than those in the developed countries [32, 33]. According to a previous study, only 39.7% of adults with diabetes achieved optimal fasting plasma glucose (FPG) control in China [34]. The Berlin acute stroke study reported that stroke patients with atrial fibrillation were associated with higher inpatient costs than those without atrial fibrillation [35]. Another Danish study demonstrated that atrial fibrillation had a 20% increase in hospital length of stay for stroke patients compared with those without atrial fibrillation [36]. In addition, a retrospective study found that patients with stroke had higher rates of diabetes, hyperlipidaemia, and hypertension [37], and the inpatient costs and LOS of patients with these three risk factors were higher than those of patients without the three risk factors.

### Unmodifiable factors and inpatient costs

In addition, our study reported that stroke subtype and sex significantly affected inpatient costs. We found that both LOS and inpatient costs were increased among patients with haemorrhagic stroke compared with those with ischaemic stroke, consistent with a 5-year follow-up study [38]. In our study, 36.94% of the patients suffered from cerebral haemorrhage, and the inpatient costs increased because of the acute onset and increased complications. In terms of sex, the inpatient costs for female patients were higher than those for male patients, which was consistent with a Korean nationwide study; females might have a longer LOS than males, and the cost of treatment was higher [39].

### Strengths and limitations of this study

Although many studies have investigated the relationship between risk factors and prevalence in adults with stroke, few have focused on the effect of risk factors on hospital costs. This retrospective study provides a new way to reduce the disease burden of stroke patients and ineffective lengths of stay. However, our study also had some limitations. First, the current study was conducted at only one hospital. The average cost of hospitalization was higher among 620 stroke patients, up to 30730.78 CNY ($ 4,455.67), which was higher than the inpatient costs of stroke patients in China in 2018, at 10213.5 CNY ($ 1,480.86) [3]. The inpatient cost of stroke patients in a single hospital may not be representative to all population in China. Second, the risk factors in our study did not take into account the effect of dietary risks and environmental risks, which also contributed to the burden of stroke. Future studies should pay more attention to the effect of those risks. Finally, the participants in our study were adults who survived a stroke event and had a high response rate. We did not have information for those who died after the stroke event, as the burden of follow-up on patients and caregivers should be underestimated.

### Implications

The findings of our study have important theoretical and practical implications for stroke prevention and reducing the disease burden of stroke patients through interventions. First, this study contributes to the literature by showing the heavy inpatient burden of adults with stroke. Chinese healthcare administrators should pay more attention to the screening and intervention of people with stroke, early detection, early diagnosis, and early treatment to control the influence of combined risk factors on inpatient costs and the reduction of the economic burden of stroke patients. Second, we found a significant effect of some modifiable factors (e.g., diabetes mellitus, atrial fibrillation, smoking, lack of physical exercise, and overweight) on LOS and inpatient costs. Community health service organizations need to guide residents to develop healthy and reasonable eating habits and lifestyles and instruct the high-risk population with these chronic diseases to enhance self-care awareness and receive regular health check-ups to avoid the occurrence of stroke or alleviate the condition of patients admitted to hospitals. Third, LOS was a significant predictor of hospital costs. To reduce inpatient costs, health policy-making departments should guide patients to seek medical treatment reasonably to reduce the LOS in higher-level hospitals for stroke rehabilitation. Finally, the mediating effect of LOS on the association between the number of risk factors and inpatient costs suggests that risk factors and LOS must be considered concurrently to reduce the inpatient burden. This mechanism provides a theoretical implication for why screening and intervention in stroke populations can reduce the medical burden.

## Conclusion

The results of this study provide strong evidence that the greater number of risk factors combined for stroke increased inpatient costs directly and indirectly by longer LOS. Compared with low-risk adults with stroke, high-risk adults incurred heavier inpatient costs due to direct or indirect effects of more length of stay. Physical inactivity had also significant direct effect and mediating effect on inpatient costs through LOS. In addition, stroke adults with smoking status, being overweight, and comorbid with diabetes mellitus and atrial fibrillation had the higher economic burden. Promoting healthy lifestyles, public education, and early screening for chronic disease should be paid more attention. The emphasis on enhancing the prevention and management of risk factors for stroke should be further reinforced.

## Data Availability

The data are available from the corresponding author upon reasonable request.

## Abbreviations

CSPPC: China Stroke Prevention Project Committee
CSHPSIP: China Stroke High-risk Population Screening and Intervention Program
TIA: Transient ischaemic attack
TIC: Total inpatient cost
VHD: Valvular heart disease
LOS: length of stay
